# Navigating mesenchymal stem cells doses and delivery routes in heart disease trials: A comprehensive overview

**DOI:** 10.1016/j.reth.2025.02.012

**Published:** 2025-03-13

**Authors:** Mohammad Reza Khalili, Salma Ahmadloo, Seyed Amin Mousavi, Mohammad Taghi Joghataei, Peiman Brouki Milan, Soheila Naderi Gharahgheshlagh, Seyedeh Lena Mohebi, Seyed Mohammad Amin Haramshahi, Vahid Hosseinpour Sarmadi

**Affiliations:** a Blood Transfusion Research Center, High Institute for Research and Education in Transfusion Medicine, Tehran, Iran; b Institute for Cognitive and Brain Science, Shahid Beheshti University, Tehran, Iran; c Department of Plastic and Reconstructive Surgery, Hazrat Fatemeh Hospital, School of Medicine, Iran University of Medical Sciences, Tehran, Iran; d Cellular and Molecular Research Center, Iran University of Medical Sciences, Tehran, Iran; e Department of Tissue Engineering and Regenerative Medicine, Faculty of Advanced Technologies in Medicine, Iran University of Medical Sciences, Tehran, Iran; f Burn Research Center, Iran University of Medical Sciences, Tehran, Iran; g Institutes of Regenerative Medicine, Faculty of Advanced Technologies in Medicine, Iran University of Medical Sciences, Tehran, Iran

**Keywords:** Mesenchymal stem cells, Heart diseases, Optimal doses, Administration routes, Clinical trial

## Abstract

In recent years, various clinical trials have been designed and implemented using mesenchymal stem cells (MSCs) for the treatment of heart diseases. Clinical trials exploring MSC-based treatments have proliferated, yet the lack of standardized protocols for MSC administration remains a significant challenge. Despite the growing popularity of MSC trials, questions persist regarding optimal dosing, administration routes, and frequency to achieve safety and efficacy, particularly in the context of cardiac regeneration. The current study has reviewed the clinical trials that have used MSCs for the treatment of heart diseases since 2009. The findings reveal diverse transplantation methods and varying MSCs quantities, highlighting the absence of a universal guideline for MSCs utilization in heart disease clinical trials.

## Graphical abstract

**Figure:**
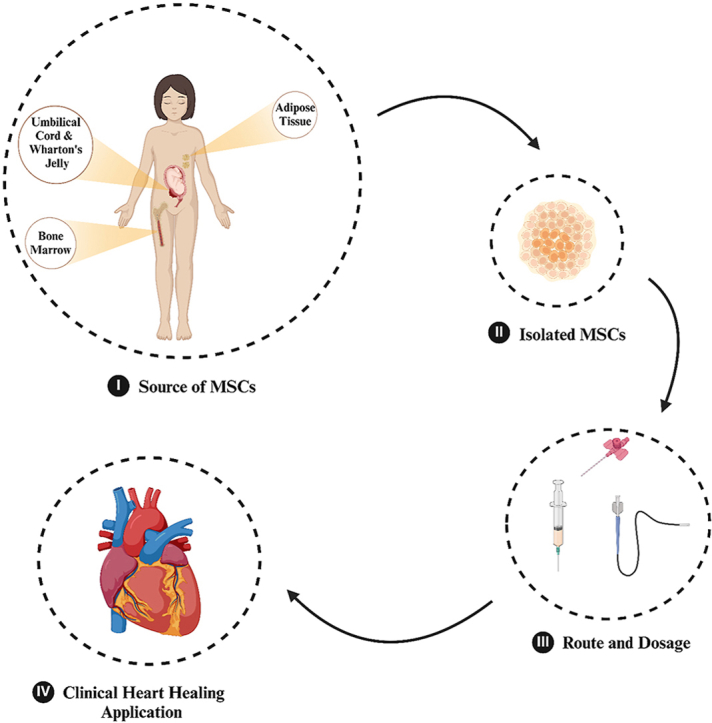

## 1 Introduction

Heart diseases consist of various conditions, such as arrhythmia, cardiomyopathy, coronary artery disease (CAD) and ischemic heart disease (IHD). Heart diseases may lead to heart failure (HF), which is one of the leading causes of long-term morbidity and mortality throughout the world [1,2]. In particular, between 3 and 5 percent of industrialized countries are affected by HF [3]. Currently, the treatment of HF is complicated and sophisticated. Surgical interventions, such as implanting mechanical ventricular assist devices, are medically high-risk and costly, while heart transplantation remains a standard curative option for end-stage HF patients. However, the scarcity of appropriate donor and life-long immunosuppression remains major hurdles for patients with end-stage HF. In addition, the low proliferative capacity of cardiomyocytes poses a challenge to the self-repair capability of the heart. (especially in the adult heart). Therefore, researchers have gradually come to grips with cell-based therapy as an advanced and alternative strategy for HF treatment [[4], [5], [6]].

In parallel with this, mesenchymal stem cells (MSCs) have been considered for cell therapy in heart diseases due to inspiring properties such as ease of access and less ethical problems. The MSCs were first characterized by Friedenstein et al. in 1970 as colony-forming unit-fibroblasts that are bone marrow-derived plastic adherent cells [7,8] and then in 1990 the term of “mesenchymal stem cells” was first used by Caplan [9]. Today, MSCs are defined by the International Society for Cell Therapy (ISCT) due to their plastic adherent capacity, cell surface markers and differentiation potential into mesodermal lineages [10]. These cells can be isolated from various sources, such as bone marrow (BM), umbilical cord blood (UCB), Wharton's jelly (WJ), and adipose-derived (AD). Presently, MSCs have fascinated researchers' consideration for clinical use due to their ease of expansion in culture, multi-lineage potential [11], providing the supportive niche for hematopoietic stem cells [12], poor immunogenicity [13,14], immunomodulatory activity [15,16], preclinical therapeutic potential [17,18], and anti-tumor activity [[19], [20], [21]]. In this respect, Kabat et al. showed the trend of MSCs clinical trials registered in ClinicalTrials.gov since 2004 in three different clinical phases. They illustrated a dramatic ascending trend in the number of clinical trials from 2008 to 2017 [22]. However, there are some obstacles which interfere with and may slow the use of MSCs in the clinic and should be tackled. One of the major barriers is the dose or number of MSCs that can be used in clinical trials. Furthermore, there is no consensus about the frequency of MSCs infusion or transplantation in the clinical trials [23,24]. Besides, the route of cell administration with the highest safety and efficacy is another key difficulty in the clinical application of MSCs. In parallel with these, the origin of MSCs' preparation protocols and MSCs passage numbers have not yet been fully specified. In summary, upon closer examination, we discover that for certain diseases, there exist clinical trials employing varying cell sources [25,26], dosages [27,28], and even distinct cell transplantation approaches. Therefore, without precise classification, researchers could become lost amidst the multitude of varied clinical trials. This study is designed to overcome some of these obstacles with a particular focus on the various hypotheses concerning the MSCs doses, frequency of doses and routes of MSCs administration in the safety and efficacy of the clinical trials which have been conducted since the last decades and aimed to uncover minimal effective doses and the optimal methods for MSCs application in heart diseases. This study seeks to provide clarity amidst the diverse landscape of clinical trials, facilitating informed decisions for future research.

## 2 Cardiac and MSCs application

The clinical application of MSCs dates back to the late 1990s, when scientists applied MSCs for a narrow range of diseases, such as bone/cartilage regeneration and cancer treatment [29,30]. Further laboratory experiments illustrated the safety and therapeutic potential of MSCs in the treatment of several diseases [31]. Specifically, in the context of heart diseases, clinical trials involving MSCs have been initiated and documented on ClinicalTrials.gov, with some completed, suspended, terminated, withdrawn, or still in recruitment [32].

Although the precise mechanism underlying MSC-mediated cardiac repair remains somewhat elusive, several plausible mechanisms have been proposed by researchers. These mechanisms consist of the promotion of paracrine signals (Hepatic growth factor (HGF), Vascular endothelial growth factor (VEGF), and Insulin-like growth factor (IGF)), stimulation of neovascularization and immunomodulation, transdifferentiation into endothelial cells and cardiomyocytes, and proliferation of endogenous cardiac stem cells with C-Kit markers [33]. Under suitable conditions both *in vivo* and *in vitro*, MSCs can differentiate into functional cardiomyocytes [34,35]. Despite similarities among MSCs from various sources, there are substantial differences in their paracrine signaling markers [36,37]. For instance, MSCs extracted from embryonic stem cells (ESC) may be a better source for neurogenic-related processes than BM-MSCs, which plummet angiogenesis in the damaged myocardium [38,39]. In addition, a study demonstrated that allogeneic MSCs can be dispensed in higher levels of nitric oxide than autologous MSCs which in turn can decrease the levels of circulating VEGF when compared to autologous MSCs. Moreover, allogeneic MSCs have been shown to be more effective than autologous MSCs in improving endothelial function in patients with heart diseases [40].

Furthermore, tumor necrosis factor alpha (TNF-α), a substantial inflammatory activator, is increased in heart-related diseases [41]. MSCs, more interestingly, also reduced the level of TNF-α in the peri-infarct myocardium [42]. In fact, both allogenic and autologous MSCs administration effectively reduce the level of TNF-α of patients with nonischemic dilated cardiomyopathy after six months of follow-up (ClinicalTrials.gov: NCT01392625) [43].

**Fig. 1:**
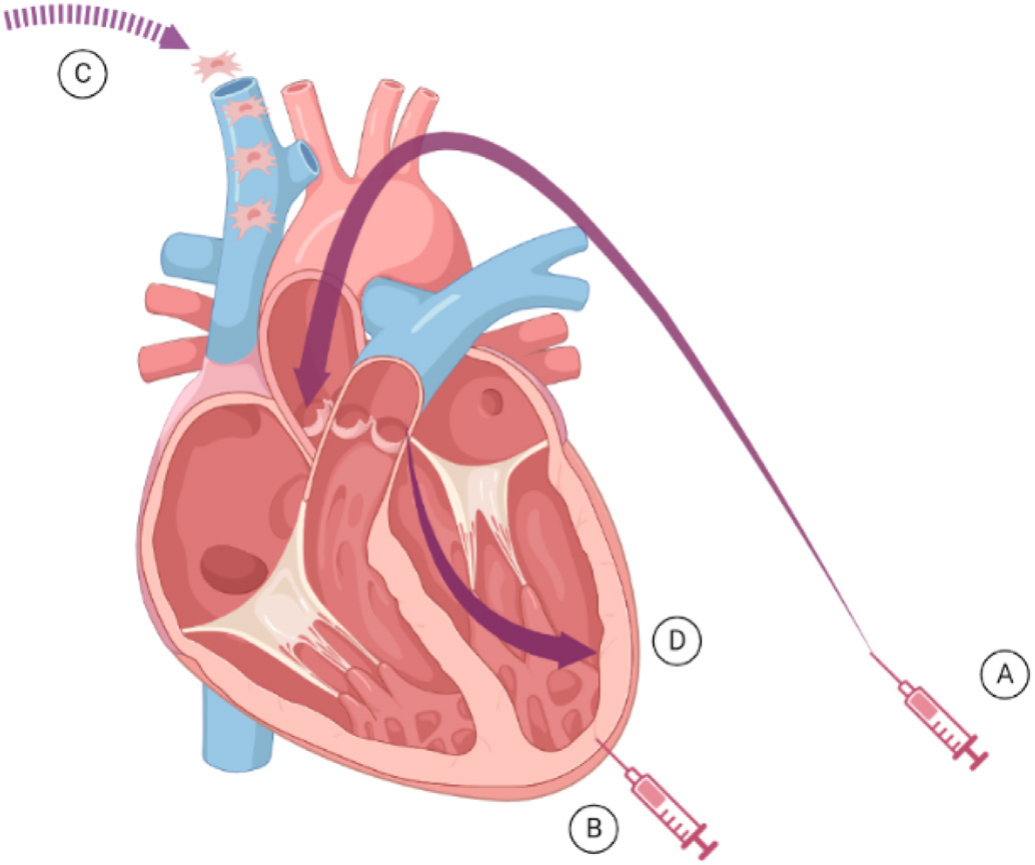
The common routs for MSCs therapy for heart tissue regeneration in clinical trials. (A) Intracoronary injection, (B) intra-myocardial injection, (C) intravenous (IV) injection, (D) trans-endocardial injection.

The route of MSCs delivery is a primordial factor that must be assessed for cardiac procedures. Currently, several promising routes have been applied for the clinical use of MSCs for heart disease, as depicted in Fig. 1, including intracoronary, intra-myocardial, intravenous (IV) and trans-endocardial injection [[44], [45], [46], [47], [48], [49]]. A systematic review by Kanelidis and colleagues illustrated that trans-endocardial injection of MSCs is more efficient than direct intra-myocardial and intracoronary injections for patients with chronic dilated cardiomyopathy and acute myocardial infarction (MI) [50]. In another study, Fakoya, demonstrated that the route of MSCs administration significantly impacts their efficacy in both acute and chronic MI [51].

### 2.1 The intracoronary delivery approaches

An intracoronary delivery system is an intervention approach for injecting MSCs into the desired zone of myocardium. This infusion is typically performed through the central lumen of a special balloon catheter fixed in the coronary artery [52]. MSCs can be released with either temporarily blocking coronary flow with balloon (minimizing rapid cell washout) or maintaining coronary flow [53].

Several clinical trials have been conducted via intracoronary injection of MSCs for heat healing applications (Table 1). Zhao et al. reported intracoronary injection of allogenic UC-MSCs in patients with chronic systolic HF could improve left ventricular ejection fraction (LVEF), 6-min walking test and mortality rate. However, their results showed one patient in the MSCs treated group out of 30, experienced chest discomfort and showed ST- and T-wave in the electrocardiogram [25]. However, spontaneous remission was achieved after 15 min. In another study, 26 patients (control group n = 12, Autologous BM-MSCs group n = 14) with acute MI investigated with intracoronary delivery cell therapy (7.2 ± 0.90 × $10^{7}$ cells). They found a significant improvement of LVEF value from baseline to the 4-month (9.0 ± 4.7 and 5.3 ± 2.6 %, p = 0.023) and 12-month (9.9 ± 5.2 % and 6.5 ± 2.7 %, p = 0.048) follow-up in the BM-MSCs group without any improvement in control group. Additionally, during the injection and follow-up periods, there was no evidence of procedural complications, life-threatening arrhythmia, or stroke [54].

**Table 1:** Roster of clinical trials have been applied intracoronary injection of MSCs for heart healing application.

Routs of Ad.	Authors/Year	Disease	Doses	No. of Pts.	MSCs Source	Outcomes	AE/SAE
Intracoronary injection	Zhang et al., 2021 [61]	Acute myocardial infarction	1.0–2.5 × $10^{6}$ cells/2 ml	43	Autologous BM-MSC	Did not identify improvement in LVEF and myocardial viability after acute MI	One death and one coronary microvascular embolism in the BM-MSCs group.
Intracoronary injection	Kim et al., 2018 [54]	Acute myocardial infarction	7.2 ± 0.90 × $10^{7}$ cells	26	Autologous BM-MSC	Improvement in LVEF observed at 12 months of follow-up.	No serious procedural complications.
Intracoronary injection	Musialek et al. 2015 [57]	Acute myocardial infarction	30 × $10^{6}$ cells	10	Allogenic WJ-MSC	No epicardial flow or myocardial perfusion impairment, and no patient showed high-sensitivity-troponin T elevation.	No AE that might be attributable to WJ-MSCs treatment.
Intracoronary injection	Gao et al. 2015 [58]	Acute myocardial infarction	6 × $10^{6}$ cells	116	Allogenic WJ-MSC	Significantly great increment in the myocardial viability and perfusion within the infarcted territory in the WJ-MSC group. Significantly great increment in the LVEF, LVESV and LVEDV in the WJ-MSC group	Infusion induced neither acute nor persistent immune or biochemical abnormalities.
Intracoronary injection	Lee et al. 2014 [55]	Acute myocardial infarction	7.2 ± 0.90 × $10^{7}$ cells	80	Autologous BM-MSC	Improvement in the LVEF.	No treatment-related toxicity and adverse cardiovascular events.
Intracoronary injection	Yang et al. 2010 [56]	Acute myocardial infarction	Two groups with different doses: Group 1: 1.22 ± 1.77 × $10^{7}$ cells group 2: 1.32 ± 1.76 × $10^{7}$ cells	16	Autologous BM-MSC	Significant improvements in LVEF, NYHA classification and myocardial viability. Non-infarct-related arteries appear safe and feasible for the treatment of patients with AMI.	No AE, arrhythmia, and any other side effects, including infections or allergic reactions.
Intracoronary injection	Zhao et al. 2015 [25]	Chronic systolic heart failure	Not mentioned (N/M)	59	Allogenic UC-MSC	Significant decrease in LVEDDs and NT-proBNP levels. Increase in LVEF. The 6-min walking test was significantly higher. Lower mortality rate.	One patient out of 30 in the MSCs group experienced chest discomfort and showed ST-T changes.
Intracoronary injection	Xiao et al. 2017 [59]	Dilated cardiomyopathy	4.9 ± 1.7 × 10^8^ cells	53	Autologous BM-MSC	Markedly improvement in LVEF, NYHA, and myocardial perfusion compared to the control group.	One AE observed hemodynamic instability that recovered within 1 h. There were no differences in the major adverse cardiovascular events between the MSCs and control groups.
Intracoronary injection	Chin et al. 2011 [60]	Severe dilated cardiomyopathy	2–3 × 10^6^ cells/kg (150 × 10^6^ cells)	5	Autologous BM-MSC	All the patients remained alive after 1 year. Significant improvements in LVEF and LVEDV were observed. Scar reduction.	No immediate post-procedural complications.

Ad.: Administration; Pts: Patients; AE: Adverse event; SAE: Severe adverse event; BM-MSCs: Bone marrow mesenchymal stem cells; MI: Myocardial infarction; LVEF: Left ventricular ejection fraction; WJ: Wharton's jelly; LVEDV: Left ventricular end-diastolic volume; LVESV: Left ventricular end-diastolic volume; NYHA: New York Heart Association; AMI: Acute myocardial infarction; UC: Umbilical cord; LVEDD: Left ventricular end-diastolic diameter; NT-proBNP: N-terminal prohormone of brain natriuretic peptide.

Intracoronary injection in either the infarct-relative artery or a non-infarct-relative artery demonstrated safety in acute MI patients along with an amelioration in LVEF, New York Heart Association (NYHA) class and myocardial viability. In a study by Lee et al. intracoronary injection of 73 × $10^{6}$ autologous BM-MSCs showed a significant increase in LVEF in MSCs treated group compared to the control group without treatment-related complication or adverse events (AE) [55]. Furthermore, Yang et al. recruited 1.22 ± 1.77 × $10^{7}$ and 1.32 ± 1.76 × $10^{7}$ autologous BM-MSCs as two separate groups with 8 patients each (total number: 16) with acute MI. Intracoronary injection in either the infarct-relative artery or a non-infarct-relative artery demonstrated the safety of non-infarct-relative artery injection in acute MI patients, along with an amelioration in LVEF, NYHA class and myocardial viability [56]. In addition, in the other two trials 6–30 × $10^{6}$ allogenic MSCs derived from WJ were injected through intracoronary in patients with acute MI. Musialek et al. showed that allogenic WJ-MSCs administration was safe without epicardial flow or myocardial perfusion impairment. Besides, Gao et al. in their safety and efficacy trial demonstrated intracoronary injection induced neither acute nor persistent immune abnormalities along with a significant elevation in the myocardial viability and perfusion within the infarcted territory in the MSCs group compare to the control. Moreover, they also noted a significant increase in LVEF and decline in left ventricular end-diastolic volume (LVESV) and left ventricular end-diastolic volume (LVEDV) in patients with acute MI [57,58].

Xiao et al.s’ trial reported hemodynamic instability as a slight AE in one patient due to MSCs therapy which it was recovered within 1 h. They applied 320–660 million autologous BM-MSCs via intracoronary routes to patients with dilated cardiomyopathy (DCM) and found that MSCs could markedly improve the LVEF, NYHA and myocardial perfusion compare to the control group [59]. Chin et al., in 2011 conducted a trial to test the feasibility and safety of intracoronary injection of autologous BM-MSCs. They demonstrated that the procedure was well tolerated by patients and there were no immediate post-procedural complications or arrhythmias. In addition, the results elucidated a significant improvement in scar reduction, LVEF and LVEDV during the 12-month follow-up period for patients with severe DCM [60].

Despite previously mentioned trials, Zhang et al., in 2021 did not identify a meaningful improvement in LVEF of patients with acute MI treated with autologous BM-MSCs via intracoronary injection. They also reported one death and one coronary microvascular embolism in the BM-MSCs group [61].

### 2.2 The intramyocardial delivery approach

Intramyocardial injection involves directly delivering MSCs into the damaged myocardial zone. This procedure usually has carried out as an adjunct during coronary artery bypass grafting. Although, this approach allows for direct visualization of the infarcted aera, it does require open-heart surgery, which carries its own risk factors [62].

**Fig. 2:**
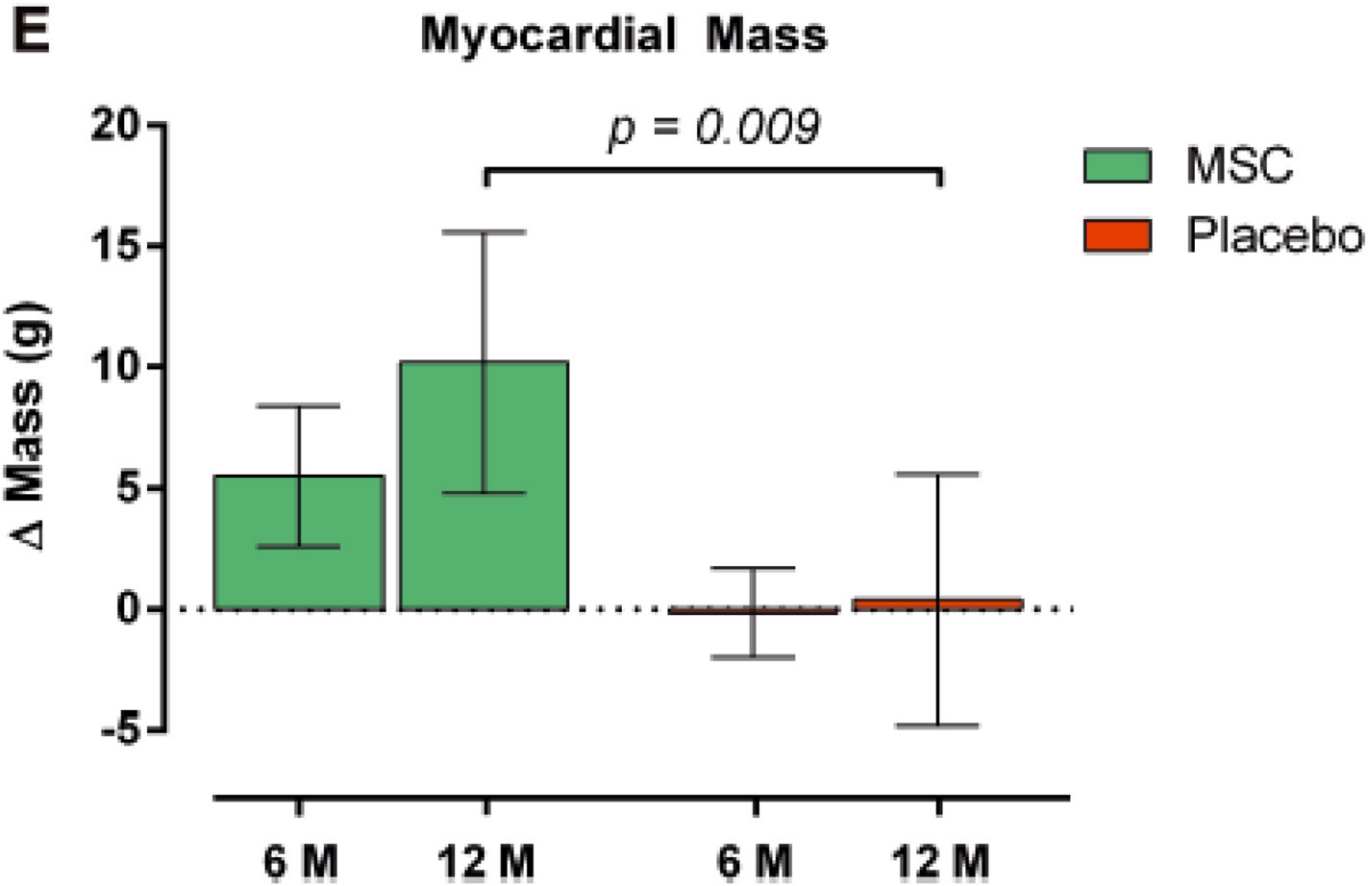
Graph of improvement in myocardial mass after 6 and 12 months of MSCs therapy via the Intra-myocardial injection compared with placebo group in the same period [64].

Table 2 delineates several clinical trials through the intramyocardial delivery approach of MSCs for the treatment of heart diseases. In terms of ischemic heart failure (IHF), intramyocardial injection of 100 × $10^{6}$ allogenic AD-MSCs significantly increased patients’ exercise capacity, LVEF, and also reduced LVEDV [63]. However, they found no sign of procedural complications or serious adverse events (SAE) related to either treatment or cell administration. In a phase 2 study diagnosed with ischemic heart failure by Mathiasen et al. two SAE related to NOGA (catheter) and injection catheters were reported. They enrolled 60 patients and injected 10–145 million autologous BM-MSCs through intra-myocardial administration. They showed significant improvement in NYHA classes, 6-min walking test, Kansas City cardiomyopathy questionnaire (KCCQ), quality-of-life score, LVEF, stroke volume (SV), cardiac output and myocardial mass (Fig. 2) [64]. In another randomized, double-blind, placebo-controlled trial, assessed 60 patients with ischemic heart failure after intramyocardial injection of BM-MSCs. They found significant reductions in the LVESV after 12 months (measured by magnetic resonance imaging or computed tomography). Moreover, there were noted significant improvements in LVEF, NYHA class, and 6-min walking test. In addition, two patients had SAE related to the NOGA procedure along with one patient with double vision and dizziness during the injection procedure [65].

**Table 2:** Roster of clinical trials have been applied intra-myocardial injection of MSCs for heart healing application.

Routs of Ad.	Authors/Year	Disease	Doses	No. of Pts.	MSCs Source	Outcomes	AE/SAE
Intra-myocardial injection	Yagyu et al. 2019 [69]	Cardiomyopathy (ischemic and nonischemic)	1.2 × $10^{7}$ to 6.5 × $10^{7}$ cells	8	Autologous BM-MSC	No significant improvement in ventricular function. No significant difference in effect on cardiac function.	During the follow-up period, there was no SAE.
Intra-myocardial injection	Karantalis et al. 2014 [66]	Ischemic cardiomyopathy	N/M	6	Autologous BM-MSC	Improvement in scar reduction, tissue perfusion, and regional function that occurs predominantly at the site of MSC injection. Increased the LVEF.	N/M
Intra-myocardial injection	Williams et al. 2011 [68]	Ischemic cardiomyopathy	100 × $10^{6}$ cells	8	Autologous BM-MSC	Decrease in end-diastolic volume, end-systolic volume and infarct size. Improved regional LV function in the infarct zone.	No patient experienced a SAE.
Intra-myocardial injection	Chin et al. 2010 [67]	Severe dilated ischemic cardiomyopathy	P: Patient P1: 28 × $10^{6}$ cells P2: 21 × $10^{6}$ cells P3: 35 × $10^{6}$ cells	3	Autologous BM-MSC	No arrhythmias were noted. Improvement in cardiac functional class and symptoms (NYHA I–II) and LVEF. Increase in muscle thickness.	N/M
Intra-myocardial injection	Chin et al. 2011 [60]	Severe dilated cardiomyopathy	0.5–1 × $10^{6}$ cells/kg (46 × $10^{6}$ cells)	5	Autologous BM-MSC	All patients remained alive at 1 year. Significant improvements in LVEF and LVEDV. Scar reduction.	No immediate post-procedural complications.
Intra-myocardial injection	Mathiasen et al., 2020 [65]	Ischemic heart failure	77.5 ± 67.9 × $10^{6}$ cells	60	Autologous BM-MSC	Improvements in NYHA, LVEF class, 6-min walking test. Reduction in LVESV. A significant increase in myocardial mass.	Two SAE about the NOGA procedure. Double vision and dizziness during the injection procedure for one patient.
Intra-myocardial injection	Kastrup et al. 2017 [63]	Ischemic heart failure	100 × $10^{6}$ cells	10	Allogenic AD-MSC	LVEDV reduction. Increase in LVEF and exercise capacity.	No complications or SAE related to either treatment or cell administration.
Intra-myocardial injection	Mathiasen et al. 2015 [64]	Ischemic heart failure	77.5 ± 67.9 × $10^{6}$ cells	60	Autologous BM-MSC	Reduction in LVESV for the MSC group. Significant improvements in NYHA class, 6-min walking test, KCCQ quality-of-life score, LVEF, stroke volume, cardiac output, and myocardial mass	Two SAE related to the NOGA procedure. No side effects were identified.
Intra-myocardial injection	Rodrigo et al. 2013 [70]	Acute myocardial infarction	31 ± 2 × $10^{6}$ cells	9	Autologous BM-MSC	The summed stress score improved. Number of ischemic segments significantly decreased. LVEV improvement.	No AE related to MSC treatment was observed during 5-year follow-up.

Ad.: Administration; Pts: Patients; AE: Adverse event; SAE: Severe adverse event; N/M: Not mentioned; BM-MSCs: Bone marrow mesenchymal stem cells; MI: Myocardial infarction; LV: Left ventricular; LVEF: Left ventricular ejection fraction; AD: Adipose; LVEDV: Left ventricular end-diastolic volume; LVESV: Left ventricular end-diastolic volume; NYHA: New York Heart Association; KCCQ: Kansas City cardiomyopathy questionnaire.

In another trial, Karantalis et al. elevated the efficacy of intra-myocardial injection of autologous BM-MSCs in patients with ischemic cardiomyopathy undergoing coronary artery bypass grafting (CABG). They showed an improvement in LVEF class, scar size, tissue perfusion and regional function, predominantly at the site of MSCs injection. The number of injected MSCs and safety reports were not specified [66].

Moreover, Chin et al., in 2010 enrolled three patients to test the feasibility and safety of intra-myocardial injection of cryopreserved autologous BM-MSCs at three various doses for each patient, 21, 28 and 35 × $10^{6}$ cells. The results demonstrated the feasibility and safety of intra-myocardial injection of cryopreserved MSCs without arrhythmias along with improvements in NYHA, LVEF and muscle thickness [67]. One year later, in 2011, his team conducted the same trial as before, but with higher number of patients and doses through two different administration routes including, intra-myocardial and intracoronary injection with relatively similar outcomes [60].

In the case of ischemic cardiomyopathy, 100 × $10^{6}$ cells autologous BM-MSCs were injected using the intra-myocardial method to analyze safety and efficacy. They showed several clinical and functional improvements without treatment-emergent SAE in any patient [68]. In contrast, Yagyu et al. did not identify significant improvements in ventricular function and LVEF after cell transplantation for both ischemic cardiomyopathy (n = 3) and nonischemic cardiomyopathy (n = 5) patients which were treated with autologous BM-MSCs. However, during the follow-up period, no participant experienced SAE such as arrhythmias [69]. In another study, patients with acute MI have also represented any AE during follow-up period following autologous BM-MSCs therapy [70].

These studies highlight the potential of intramyocardial MSCs therapy for ischemic cardiomyopathy, emphasizing the need for careful consideration of cell quantity and safety aspects. Continued research will refine our understanding and optimize MSCs-based treatments for cardiac regeneration.

### 2.3 The intravenous (IV) delivery approach

The intravenous (IV) is the most straightforward cell delivery method for the treatment of heart diseases. This approach has been deployed through central venous or peripheral catheters to slump interventional setbacks. However, because MSCs are dispersed in other internal organs such as the spleen, liver, and lungs, the IV delivery approach is less efficient than other methods for treating heart diseases [71].

In the case of nonischemic cardiomyopathy trials, in one clinical study, 22 patients were enrolled to test the safety and efficacy of 1.5 × $10^{6}$ allogenic BM-MSCs per kilogram through IV administration. They realized that MSCs therapy caused an immunomodulatory effect, along with one AE, and bruising at the IV infusion site. Furthermore, they showed significant reductions in LVEDV and LVESV and improvement in LVEF in the 6-min walking test, KCCQ and NYHA classification in allogenic BM-MSCs treated patients [45].

Furthermore, another trial was conducted in patients with MI by using the IV method. In this regard, Hare et al. enrolled 53 patients with MI by using three doses escalation group (0.5, 1.6 and 5 × $10^{6}$ cells/kg) to evaluate the safety and efficacy of allogenic BM-MSCs via IV administration. They found improvements in LVEF, pulmonary function, cardiac performance, and EF without AE related to study treatment [72].

The safety and efficacy of IV administration of allogenic UC-MSCs have also been evaluated in a randomized, double blind, placebo-controlled clinical trial in patients with chronic stable HF and reduced EF. The results illustrated significant improvements in LVEF, LVEDV, NYHA class, Minnesota Living with Heart Failure Questionnaire (MLHFQ), KCCQ and ventilation/volume of exhaled carbon dioxide (VE/V ${CO}_{2}$) with no acute AE associated with the infusion of MSCs [73]. However, another trial applied allogenic UC-MSCs via IV injection for patients with congestive heart failure at a dosage of 50–100 × $10^{6\;}$ cells. Although an increase of LVEF was observed in two patients, one patient experienced a significant decrease in LVEF. In addition, all patients illustrated remarkable improvement in NYHA (Table 3) [26].

**Table 3:** Roster of clinical trials have been applied IV injection of MSCs for heart healing application.

Routs of Ad.	Authors/Year	Disease	Doses	No. of Pts.	MSCs Source	Outcomes	AE/SAE
IV injection	Bartolucci et al. 2017 [73]	Heart failure	1 × $10^{6}$ cells/kg	12	Allogenic UC-MSC	Significant improvements in LVEF, LVEDV, NYHA class, Minnesota Living with heart failure questionnaire, KCCQ, and VE/V ${CO}_{2}$.	No acute AE associated with the infusion of MSCs.
IV injection	Fang et al. 2016 [26]	Congestive heart failure	50–100 × $10^{6}$ cells	3	Allogenic UC-MSC	Increase LVEF in two patients and decrease in one patient. Significant improvement in 6-min walking test in two patients and NYHA of all patients.	There was no SAE.
IV injection	Butler et al. 2017 [45]	Nonischemic cardiomyopathy	1.5 × $10^{6}$ cells/kg	22	Allogenic BM-MSC	Significant reduction in LVEDV, and LVESV. Increase in LVEF, 6-min walking test, and improved in KCCQ and NYHA.	No SAE. One MSCs-related AE related to bruising at the IV infusion site
IV injection	Hare et al. 2009 [72]	Myocardial infarction	Three doses escalation groups 0.5, 1.6, and 5 × $10^{6}$ cells/kg	53	Allogenic BM-MSC	Improvements in LVEF, pulmonary function, cardiac performance, global symptom score and ejection fraction	No AE was considered to have a probable relation to the study treatment.

Ad.: Administration; Pts: Patients; AE: Adverse event; SAE: Severe adverse event; UC: Umbilical cord; BM-MSCs: Bone marrow mesenchymal stem cells; LVEF: Left ventricular ejection fraction; LVEDV: Left ventricular end-diastolic volume; LVESV: Left ventricular end-diastolic volume; NYHA: New York Heart Association; KCCQ: Kansas City cardiomyopathy questionnaire; VE/V ${CO}_{2}$: Ventilation/volume of exhaled carbon dioxide (VE/V ${CO}_{2}$).

### 2.4 The trans-endocardial delivery approach

The trans-endocardial delivery approach is a minimally interventional approach for delivering MSCs or other therapeutic agents directly into the myocardium. This procedure is typically performed using a needle-tipped catheter inserted through a peripheral artery and led across the aortic valve [74]. Some studies suggest that trans-endocardial administration offers higher MSCs maintenance compared to intracoronary and IV injections [75,76].

Several clinical trials have been conducted on MSCs therapy for heart diseases via trans-endocardial injection (Table 4). In one study, 30 patients were enrolled to test the safety and efficacy of trans-endocardial injection of MSCs. They applied two doses in two separate groups with 20 million and 100 million allogenic BM-MSCs. They illustrated the safety and feasibility of trans-endocardial stem cell injection for both treatment groups. Additionally, it showed that MSCs could improve cardiac function, with an increase in EF observed in high-dose group and reduction in scar size in both groups. Furthermore, the results showed improvement in the 6-min walking test and NYHA class, with the 100 million group imparting greater benefit [77]. In another study, Heldman et al. injected autologous BM-MSCs (200 × $10^{6}$ cells) into patients with ischemic cardiomyopathy. Over a 12-mounth follow-up, improvements were observed in LV chamber volume, EF, MLHFQ score, and the 6-min walking test, with no SAE reported. However, regional myocardial activity, as measured by peak Eulerian circumferential strain at the zone of administration, improved with MSCs but not in the placebo group [78].

**Table 4:** Roster of clinical trials have been applied trans-endocardial injection of MSCs for heart healing application.

Routs of Ad.	Authors/Year	Disease	Doses	No. of Pts.	MSCs Source	Outcomes	AE/SAE
Trans-endocardial injection	Florea et al., 2020 [79]	Non-ischemic dilated cardiomyopathy	N/M	34	Allogenic and autologous BM-MSC	Improvements in NYHA, MLHFQ class, 6-min walking test and cardiac function Serum TNF-α levels decreased. Improvement of. Endothelial function in both sexes.	N/M
Trans-endocardial injection	Hare et al. 2017 [43]	Non-ischemic dilated cardiomyopathy	100 × $10^{6}$ cells	37	Allogenic and autologous BM-MSC	Greater magnitude and clinically meaningful effects in allogenic than autologous MSCs, including significant improvement in EF, 6-min walking test, MLHFQ scores, endothelial function, and NYHA class. Greater TNF-α suppression.	Lower rate in post- trans-endocardial injection SAE, rehospitalization rate and major adverse cardiovascular event in the allogenic group than the autologous group.
Trans-endocardial injection	Florea et al. 2017 [77]	ICM	Two groups: Group 1: 20 × $10^{6}$ Group 2: 100 × $10^{6}$ cells	30	Allogenic BM-MSC	Cardiac function improvement. Scar size reduction in both groups and the EF improvement only with 100 million cells. Increase in proBNP in 20 million cells, but not in 100 million cells. Improvement in 6-min walk test, NYHA class and infarct size. The higher dose is superior to the lower dose.	No treatment-related serious AE at 12 months.
Trans-endocardial injection	Heldman et al. 2014 [78]	ICM	200 × $10^{6}$ cells	30	Autologous BM-MSC	Improvement in the MLHFQ score over 1 year. Increased in the 6-min walking test in the MSCs group. Reduction in infarct size by MSCs. Improvement in regional myocardial function as peak Eulerian circumferential strain at the site of injection.	No treatment-emergent SAE among any of the patients.
Trans-endocardial injection	Hare et al. 2012 [44]	ICM	20 × $10^{6}$ 100 × $10^{6}$ 200 × $10^{6}$ cells There are two groups: Either an allogenic or an autologous group, and each group receives three increasing dose levels.	30	Allogenic and autologous BM-MSC	Improvement in the 6-min walk test and the MLHFQ score in both groups, but not significant in the allogeneic group. Improvement in NYHA class 50 % and 28.6 % in the autologous and allogenic group, respectively. Both groups reduced the mean EED and LV sphericity index. Reduction in LVEDV only in the allogeneic group. Low-dose of MSCs produced the greatest reductions in LV volumes and increased EF.	Each group has one treatment-emergent SAE. 6 and 17 AE in the allogeneic and autologous group in 30 days, respectively. Over 12 months, one SAE in 5 and 8 patients and 24 and 38 AE in the allogeneic and autologous group, respectively. No ventricular arrhythmia SAEs in the allogeneic group compared with 4 patients in the autologous group at 1 year.
Trans-endocardial injection	Premer et al. 2015 [40]	Heart failure due to either idiopathic DCM or ICM	DCM: Either allogenic or autologous 100 × $10^{6}$ cells ICM: allogenic Either 20 or 100 × $10^{6}$ cells	22	Allogenic and autologous BM-MSC	Improvement in EPC-CFUs, FMD% and endothelial function in the allogeneic MSCs group not autologous group. Reduction in serum VEGF level in the allogenic group and increase in the autologous group.	N/M

Ad.: Administration; Pts: Patients; AE: Adverse event; SAE: Severe adverse event; N/M: Not mentioned; BM-MSCs: Bone marrow mesenchymal stem cells; NYHA: New York Heart Association; MLHFQ: Minnesota Living with Heart Failure Questionnaire; TNF-α: Tumor necrosis factor alpha; NT-proBNP: N-terminal prohormone of brain natriuretic peptide; LV: Left ventricular; EED: end-diastolic diameter; EF: Ejection fraction; LVEDV: Left ventricular end-diastolic volume; EPC-CFUs: Endothelial progenitor cell-colony forming units; FMD: flow-mediated vasodilation; VEGF: Vascular endothelial growth factor; DCM: Dilated cardiomyopathy; ICM: Ischemic cardiomyopathy.

In other remarkable study by Hare et al. on ischemic dilated cardiomyopathy, they used trans-endocardial injection of autologous or allogenic BM-MSCs in the two groups. Each group was then divided into three subgroups according to the dose escalation (20, 100, and 200 million cells). The results showed significant ameliorate in the 6-min walking test, MLHFQ and NYHA classes in the autologous group, but not in the allogenic group. Furthermore, both groups had reduced mean end-diastolic diameter (EED) and LV sphericity index in the patients although, LVEDN reduction just occurred in the allogenic group. Moreover, they illustrated that a low dose of MSCs (20 million cells) could produce the greatest reduction in LV volumes and increase in EF. The findings from this study demonstrate that an increased cell count does not invariably result in improved outcomes. Indeed, the quantity of cells can significantly influence the obtained results and requires meticulous consideration, particularly in light of the study's specific type. Finally, their results showed one treatment-emergent SAE in each group and few AE, which were greater in the autologous group, within 30 days and 1-year follow-up [44].

To analyze the changes in endothelial function via trans-endocardial injection, Premer et al. enrolled 22 patients with HF due to either idiopathic DCM or ischemic cardiomyopathy (ICM). They measured endothelial progenitor cell-colony forming units (EPC-CFUs) and flow-mediated vasodilation (FMD) after administering 20–100 × $10^{6}$ allogeneic or autologous BM-MSCs. The study found an improvement in EPC-CFUs and the percent of FMD in the allogenic and autologous MSCs groups, and consequently improved endothelial function in the allogenic group [40].

However, in a trial with 24 male and 10 female patients with non-ischemic DCM who were treated via trans-endocardial injection of allogenic and autologous BM-MSCs, both sexes experienced improvements in the MLHFQ, the 6-min walking test, and NYHA. Furthermore, after 12-monthe follow-up, the levels of TNF-α and EPC-CFUs significantly improved in both male and female patients [79]. In the same study, even though allogenic and autologous MSCs improved the functional index of the heart in patients with nonischemic DCM, they illustrated that allogenic MSCs were more effective than autologous MSCs in improving EF, the 6-min walking test, MLHFQ, and TNF-α suppression. More interestingly, the number of hospitalized patients with SAE in the allogenic group was lower than that in the autologous [43].

## 3 Future prospective

Enormous independent, high-quality clinical trials related to the applications of MSCs have taken a significant step toward treating or improving a wide spectrum of heart diseases. Even though all of these clinical studies have demonstrated the efficacy and safety of MSCs therapy, there are enormous limitations and unresolved issues that need to be addressed.

First and foremost, the exact mechanism of the biology and molecular elements of MSCs remains ambiguous. Needless to say, understanding the biology and role of the different types of MSCs is necessary to purify their manufacturing process and maximize their capacity to promote tissue repair. In particular, it is important to compare different types of MSCs from various tissue sources (e.g., BM, AD, UCB, and perinatal) in terms of cardiac regenerative properties. Additionally, exploring the inner and outer signaling pathways (such as cell differentiation and proliferation) of MSCs will lead researcher to apply these cells in efficient applications.

Second, the route of MSCs delivery is another factor that needs to be limited. Optimizing the MSCs delivery method can enhance safety and efficacy, especially for heart diseases. In this case, the trans-endocardial route indeed appears promising for efficient MSCs administration compared to systemic circulation delivery [50].

Finally, with the gaining of in-depth knowledge about MSCs and their combination with bioengineering scope, coining and developing the new methods for application of MSCs applications are not far-fetched destinations. According to this, numerous intricate methods such as genetic modification for cardiac regeneration [80], pre-conditioning agents [81,82], and MSCs pretreatment factors (e.g., basic FGF and IGF-1 [83]) are being explored. Developing novel delivery methods and targeted blockage of MSCs in affected myocardial zones is an exciting avenue [[84], [85], [86]]. Therefore, continued research and collaboration will uncover further insights and propel MSC-based treatments toward better outcomes for patients with heart diseases.

## Authors' contributions

M.R.K: Conceptualization, Original draft; V.H.S and S.M.A.H: Draft Preparation, Writing, Review, Editing, Supervision and Project Administration; S.A, M.T.J, P.B.M, S.N.G, G.L and S.L.M: Draft Preparation and Data Collection.

## Declaration of competing interest

The authors declare that they have no known competing financial interests or personal relationships that could have appeared to influence the work reported in this paper.
